# Repositioning candidate gene approaches in the post-GWAS era: a clinical perspective from psychiatric pharmacogenetics

**DOI:** 10.3389/fgene.2026.1828563

**Published:** 2026-08-18

**Authors:** Milica Pjevac, Jurij Bon, Vita Dolžan

**Affiliations:** 1 University Psychiatric Clinic, Center for Clinical Psychiatry, Ljubljana, Slovenia; 2 Department of Psychiatry, Faculty of Medicine, University of Ljubljana, Ljubljana, Slovenia; 3 Pharmacogenetics Laboratory, Faculty of Medicine, Institute of Biochemistry and Molecular Genetics, University of Ljubljana, Ljubljana, Slovenia

**Keywords:** candidate gene studies, GWAS, pharmacogenomics, pharmacogenetics, precision psychiatry

## Abstract

The clinical adoption of pharmacogenetics has advanced unevenly despite rapid progress in genomic discovery. Genome-wide association studies and next-generation sequencing have substantially improved our understanding of the polygenic and regulatory architecture of complex diseases, yet translating these insights into clinically actionable pharmacogenetic strategies remains challenging. In this opinion article, we argue that targeted genetic approaches, traditionally represented by candidate gene association studies, should be reconsidered within a modern genomic framework. While such approaches proved limited as discovery tools for complex disease risk, they may retain translational value when integrated with genome-wide evidence and multi-omics prioritization. In pharmacogenetics, pathway-focused investigations and targeted validation studies can help bridge the gap between large-scale genomic discovery and clinical implementation. Within this framework, pharmacokinetic pharmacogenes (e.g., CYP2D6, CYP2C19) and pharmacodynamic phenotypes (e.g., treatment response, antipsychotic-induced weight gain) represent fundamentally different translational challenges, differing in effect size, biological complexity, and clinical readiness. We propose that the strategic integration of genome-wide analyses, sequencing, multi-omics data, and hypothesis-driven genetic studies may accelerate the clinical adoption of pharmacogenetics and contribute to more effective precision medicine in psychiatry.

## Introduction

The past 3 decades have witnessed a fundamental transformation in the study of complex disease genetics. The advent of genome-wide association studies (GWAS) and next-generation sequencing has moved psychiatric genetics from small-scale, hypothesis-driven investigations toward large-scale, hypothesis-free discovery. These advances have substantially expanded our understanding of the polygenic architecture underlying schizophrenia, major depressive disorder, bipolar disorder, and related conditions, implicating thousands of common variants of individually small effect and revealing the distributed, regulatory nature of genetic risk (International Schizophrenia Consortium et al., 2009; Andreassen et al., 2023). Yet the accumulation of genomic discovery has not translated proportionally into clinical progress. The gap between statistical association and actionable clinical insight remains wide, and in psychiatry, it is wider than in almost any other medical specialty.

Psychiatry occupies a distinctive position within this landscape. Unlike many somatic conditions, psychiatric disorders lack objective biomarkers for diagnosis, treatment selection, or therapeutic monitoring. The clinical burden of this uncertainty is substantial: antidepressant non-response rates approach 50% in real-world settings (Rush et al., 2006), and antipsychotic-induced metabolic complications are a leading cause of premature cardiovascular mortality in patients with serious mental illness (De Hert et al., 2012; Scigliano and Ronchetti, 2013). The human and economic costs of the prevailing trial-and-error model in psychopharmacotherapy are therefore considerable (Arnaud et al., 2021).

Within psychiatric genetics, two broad but distinct domains of inquiry have developed in parallel. Disease-risk genetics seeks to identify genetic variants that increase susceptibility to psychiatric disorders. Pharmacogenetics and pharmacogenomics, by contrast, investigate how genetic variation shapes drug response: metabolism, receptor sensitivity, efficacy, and adverse effect profiles. Throughout this article, ‘pharmacogenetics’ refers to the study of how variation in specific genes influences drug response, and ‘pharmacogenomics’ refers to broader genome-scale and multi-omics approaches to the same question; where the distinction is not critical, the terms are used interchangeably in accordance with common practice in the field. Pharmacogenetic phenotypes are more proximate to molecular mechanisms, less polygenic, and more amenable to clinical operationalisation than psychiatric diagnoses themselves (Fabbri, 2022; Linskey et al., 2021). Within pharmacogenetics itself, a further distinction is clinically consequential: pharmacokinetic markers, such as drug-metabolising enzyme variants (CYP2D6, CYP2C19), tend to exert larger, more mechanistically tractable effects amenable to single-gene clinical operationalisation; pharmacodynamic phenotypes—including antidepressant treatment response and antipsychotic-induced metabolic effects—typically involve smaller effect sizes, greater polygenicity, and considerably more complex biological pathways, placing them at an earlier stage of translational readiness. The relevance of pharmacogenetics extends well beyond psychiatry: the field has already achieved actionable clinical implementation in oncology, cardiology, and rheumatology, and those trajectories provide an instructive reference point for understanding both the potential and the current limitations of psychiatric pharmacogenetics. Examining how genetic analyses were repositioned in these specialties and what advantages this brought is, therefore, both scientifically informative and practically relevant for advancing precision psychiatry.

Yet despite this promise, the translation of pharmacogenetic and pharmacogenomic findings into routine psychiatric practice remains strikingly incomplete (Bousman et al., 2021). While prior contributions have argued for shifting pharmacogenetic research toward GWAS-first frameworks (Linskey et al., 2021) or have reviewed genetic methods in psychiatry broadly (Fabbri, 2022; Trkulja, 2024), a comparative cross-specialty analysis situating psychiatry’s trajectory relative to oncology, cardiology, and rheumatology has not been systematically undertaken. The present article addresses this gap by proposing a cross-specialty translational framework, criteria for second-generation candidate studies, and an organisational model for clinical implementation. Despite the establishment of actionable guidelines for a subset of well-validated gene-drug pairs by bodies such as the Clinical Pharmacogenetics Implementation Consortium (CPIC), uptake remains uneven and the proportion of prescribing decisions informed by genetic testing is low. The reasons are both scientific and organisational: gaps in the evidence base, methodological heterogeneity, structural complexity of key pharmacogenes such as CYP2D6 and CYP2C19, and the absence of integrated pathways linking genomic research to clinical decision-making (Fabbri, 2022; Linskey et al., 2021; Bousman et al., 2021).

## Pharmacogenetics across medical specialties: lessons for psychiatry

The repositioning of genetic approaches in psychiatry is best understood against the backdrop of parallel developments in other medical specialties. Oncology, cardiology, and rheumatology have each navigated the transition from early candidate-gene reasoning to genome-informed, clinically implemented pharmacogenetics, and the structural features that enabled implementation in those fields are directly relevant for psychiatry.

## Oncology: a template for companion diagnostics and toxicity prevention

Oncology has achieved the most advanced integration of pharmacogenomics into routine clinical care of any medical specialty, offering two distinct implementation paradigms. The first is tumour companion diagnostics: genomic characterisation of a tumour determines treatment eligibility. HER2 overexpression testing before trastuzumab therapy in breast cancer was among the first such applications; BRCA1/2 germline testing before PARP inhibitor therapy (olaparib, niraparib) and EGFR/ALK testing before tyrosine kinase inhibitor prescription followed the same logic. In each case, a candidate gene hypothesis (biologically obvious target) was validated at scale and then embedded as a regulatory requirement for drug prescribing (Nogueiras-Álvarez and Pérez Francisco, 2024). The psychiatric equivalent would be a validated pharmacogenetic test required before initiating a specific psychotropic, a standard not yet achieved but toward which the field is moving.

The second oncology paradigm, germline pharmacogenetics for toxicity prevention, is more directly analogous to psychiatric pharmacogenetics. DPYD genotyping before fluoropyrimidine chemotherapy (5-fluorouracil, capecitabine) identifies patients with dihydropyrimidine dehydrogenase deficiency who face life-threatening toxicity at standard doses; CPIC-mandated dose reductions in DPYD-deficient patients have substantially reduced severe and fatal adverse events (Maslarinou et al., 2023; Koutsilieri and Patrinos, 2020). Similarly, TPMT (thiopurine S-methyltransferase) and NUDT15 (nudix hydrolase 15) genotyping before thiopurine therapy prevents myelosuppression in enzyme-deficient patients; this is now the preemptive standard-of-care in paediatric acute lymphoblastic leukaemia (ALL), where pharmacogenetics-guided thiopurine dosing has contributed to 5-year survival rates exceeding 90% (Relling and Evans, 2015; Moriyama et al., 2016).

The structural features that enabled oncological pharmacogenetics to achieve clinical implementation are identifiable and replicable: first, each successful application centred on a single, mechanistically understood gene-drug pair with a large and clinically consequential effect size; second, the transition from candidate hypothesis to genome-wide confirmation to prospective clinical validation followed a structured sequential pipeline; and third, guideline bodies formalised evidence into prescribing recommendations. Psychiatric pharmacogenetics has completed this pipeline for CYP2D6 and CYP2C19 in antidepressant metabolism, and the DPYD/TPMT experience demonstrates that the same approach is directly applicable to adverse effect prevention—precisely the challenge posed by antipsychotic-induced metabolic toxicity.

## Cardiology: point-of-care genotyping and shared molecular actors

Cardiovascular medicine offers a second revealing comparison, and one with a striking molecular overlap with psychiatry. Clopidogrel, the antiplatelet pro-drug most widely used after percutaneous coronary intervention (PCI), requires CYP2C19-mediated bioactivation. Loss-of-function CYP2C19 alleles carried by 20%–50% of certain populations attenuate platelet inhibition and significantly increase the risk of stent thrombosis and major adverse cardiovascular events (MACE) (Scott et al., 2013; Sorich et al., 2014). The POPular Genetics trial subsequently demonstrated that CYP2C19-guided antiplatelet therapy substituting ticagrelor or prasugrel for clopidogrel in poor/intermediate metabolizers reduced MACE without increasing bleeding (Claassens et al., 2019). CYP2C19 genotyping is now recommended by the American Heart Association and NICE (UK) for patients undergoing PCI (Patel et al., 2025).

The cardiology trajectory is directly relevant to psychiatric pharmacogenetics because the central molecular actor is the same: CYP2C19. The same gene whose poor-metaboliser alleles predict clopidogrel non-activation in cardiology predicts antidepressant accumulation, adverse drug reactions, and remission rates in psychiatry. This overlap is not coincidental, and it carries a direct practical consequence: the evidentiary and regulatory infrastructure built for CYP2C19-guided prescribing in cardiology directly strengthens the case for its adoption in psychiatry. The POPular Genetics trial model—a prospective, genotype-guided randomised trial with a clinical endpoint—is precisely the study design that psychiatric pharmacogenetics now needs for its own CYP2C19-related interventions.

Warfarin pharmacogenetics provides a parallel example: CYP2C9 and VKORC1 variants together account for 30%–60% of the variance in stable warfarin dose requirements, and pharmacogenetic dosing information has been incorporated into the FDA drug label. The warfarin story exemplifies the same sequential logic: a candidate gene hypothesis, identified through biochemical reasoning, progressively validated through population studies, GWAS fine-mapping, and prospective clinical trials before guideline incorporation (Kimmel et al., 2013; Shorbaji et al., 2025).

## Rheumatology: an analogous stage of development

Rheumatology presents a third comparator, currently at an earlier stage of pharmacogenetic implementation than oncology or cardiology, and therefore perhaps the most directly analogous to psychiatry. Pharmacogenetic models combining polymorphisms in MTX-pathway genes with clinical variables have been validated in several cohorts, though none are yet embedded in routine prescribing guidelines (Wessels et al., 2007; Fransen et al., 2012). TNF inhibitors achieve a good response in only approximately 60% of patients, and GWAS-identified predictors still require external validation before clinical implementation (Curry et al., 2023). Rheumatology thus mirrors psychiatry in its current challenges: phenotype heterogeneity, modest effect sizes, and absence of validated pharmacogenetic tests for its most important biologics. The TPMT story illustrates that pharmacogenetic implementation can generalise across specialties once the evidential standard is met: when a pharmacogenetic evidence base matures in one specialty, the regulatory and clinical infrastructure can be adapted elsewhere.

Table 1 summarises the stage of pharmacogenetic implementation across the four specialties discussed.

**TABLE 1:** Pharmacogenetic implementation across medical specialties, with relevance for psychiatry.

Specialty	Key gene-drug pair	Implementation stage & key enablers	Lessons for psychiatry
Oncology (toxicity PGx)	DPYD – 5-fluorouracil; TPMT/NUDT15 – thiopurines	Fully implemented; CPIC mandatory; preemptive genotyping standard. Enabled by: single gene-drug pair, large effect size, clear toxicity endpoint, prospective RCT validation	Toxicity-prevention model; sequential evidence pipeline; EHR-embedded decision support
Oncology (companion Dx)	HER2 – trastuzumab; BRCA1/2 – PARP inhibitors; EGFR – erlotinib	Regulatory requirement; genotype required before Rx. Enabled by: tumour-specific target, clinical trial integration, FDA companion Dx approval	Aspirational model for biomarker-guided Rx in psychiatry
Cardiology	CYP2C19 – clopidogrel; CYP2C9/VKORC1 – warfarin	Implemented; FDA label + CPIC; AHA/NICE guidelines; point-of-care genotyping. Enabled by: shared CYP2C19 gene, prospective RCT (POPular genetics), clear outcome (MACE)	Direct CYP2C19 overlap; POPular genetics as RCT model; regulatory pathway established
Rheumatology	MTHFR/ATIC – methotrexate; FCGR2A – TNF inhibitors; TPMT – azathioprine	Partial; TPMT standard; MTX/TNFi models validated but not routine. Challenges: Heterogeneous phenotypes, modest effect sizes	Most analogous current stage; TPMT cross-specialty transfer demonstrates generalisability
Psychiatry	CYP2D6/CYP2C19 – antidepressants (SSRIs, SNRIs); MC4R/HTR2C – antipsychotic weight gain; PDE4B – substance use	Partial; CPIC guidelines for SSRI/SNRI dosing; broader pipeline developing. Challenges: no diagnostic biomarkers, polygenic outcomes	CYP2D6/CYP2C19 evidence base the most mature in psychiatry; CPIC guidelines established but real-world implementation remains variable and clinical utility continues to be evaluated. AIWG: no validated clinical test exists, prospective multi-cohort replication required. PDE4B/substance use: early-stage, pipeline incomplete

Abbreviations: MACE, major adverse cardiovascular events; Dx, diagnostics; ALL, acute lymphoblastic leukaemia. Effect sizes in footnote: DPYD deficiency: 2–4× increased severe fluoropyrimidine toxicity risk (Maslarinou et al., 2023; Koutsilieri and Patrinos, 2020; Gmeiner, 2021); POPular Genetics: HR 0.78 (95% CI, 0.61–0.98) for PLATO major/minor bleeding (Claassens et al., 2019); thiopurine dosing: >90% 5-year survival in paediatric ALL (Relling and Evans, 2015; Moriyama et al., 2016).

## Limitations of early candidate gene approaches

Early molecular genetic studies in psychiatry were guided by biological plausibility. Variants in genes encoding neurotransmitter receptors, transporters, and metabolic enzymes, particularly in the dopaminergic and serotonergic systems, were tested against prevailing neurochemical models of psychiatric illness (Varga et al., 2011). Conceptually coherent, this strategy proved empirically fragile. Many reported associations failed to replicate, likely due to small sample sizes, inflated effect estimates, population stratification, and publication bias (Johnson et al., 2017; Farrell et al., 2015). More fundamentally, these studies assumed that common variants within a limited set of biologically obvious genes would exert moderate effects on disease risk (Ioannidis, 2008).

GWAS decisively challenged this assumption. Large-scale analyses demonstrated that psychiatric disorders are highly polygenic, shaped by thousands of common variants of individually small effect (Schizophrenia Working Group of the Psychiatric Genomics Consortium et al., 2014; Wray et al., 2018). Genetic liability is distributed across the genome rather than concentrated in a handful of core genes, replacing gene-centric models with distributed regulatory architectures (Boyle et al., 2017; Visscher et al., 2017).

A conceptually important distinction concerns the inferential status of contemporary targeted pharmacogenetic studies. First-generation candidate gene investigations were fundamentally exploratory: they generated hypotheses from neurobiological reasoning and tested them in the same dataset, without robust prior evidence—a structural driver of the reproducibility crisis (Border et al., 2019; Munafò et al., 2017). A repositioned second-generation approach is confirmatory rather than exploratory: it tests pre-specified hypotheses already supported by independent genome-wide or multi-omics evidence (Trkulja, 2024; Nosek et al., 2018). This distinction parallels the evolution seen in oncology and cardiology, where early candidate gene findings (CYP2D6 in antidepressant metabolism; CYP2C19 in clopidogrel activation; DPYD in fluoropyrimidine toxicity) were initially inconsistent precisely because they were exploratory and underpowered, achieving clinical utility only after prospective confirmatory validation in adequately powered cohorts. The lesson from those fields is not that candidate gene approaches are inherently flawed, but that their evidential standard must be fundamentally different from the first generation.

## Criteria for a rigorous second-generation candidate gene study

A contemporary candidate gene study in psychiatric pharmacogenetics must satisfy several minimum criteria. First, candidate selection must be externally justified by prior GWAS signals, eQTL co-localisation, or convergent multi-omics evidence, not biological plausibility alone (Giambartolomei et al., 2014; Qi et al., 2024). Second, the hypothesis and analysis plan must be pre-specified before data collection, ideally through prospective registration (Border et al., 2019; Simmons et al., 2011). Third, statistical power must be calculated *a priori* using realistic effect sizes from prior genome-wide data for pharmacogenetic phenotypes with moderate effect sizes, typically requiring several hundred to over a thousand participants (Sham and Purcell, 2014). Fourth, correction for multiple comparisons must be applied. Fifth, replication in an independent cohort must be a condition of inference, not a post-publication aspiration (Trkulja, 2024). Sixth, functional characterisation—eQTL data, splicing analyses, or *in vitro* validation—should accompany the clinical association (Wainberg et al., 2019).

## Illustrative examples of the translational pipeline

**FIGURE 1:**
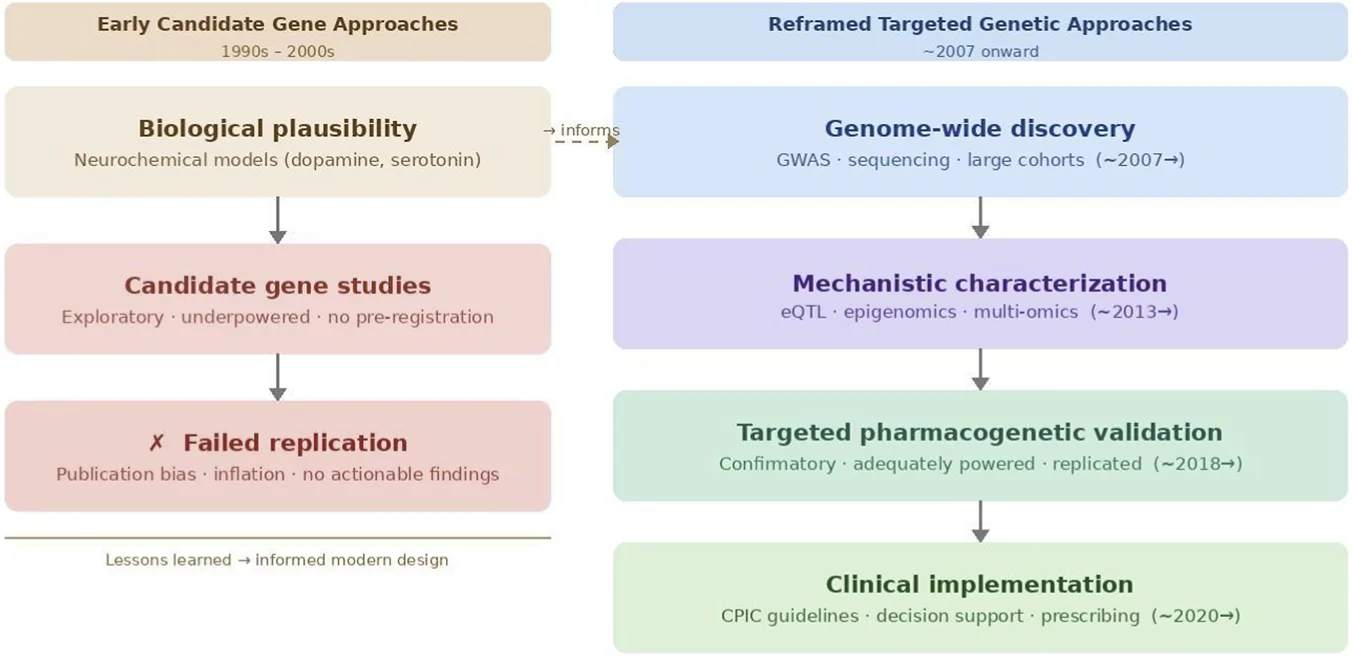
From candidate gene studies to precision psychiatry: a repositioned translational pipeline.

A foundational distinction must be drawn between two structurally different contexts within this pipeline. Pharmacokinetic pharmacogenes such as CYP2D6 and CYP2C19 are mechanistically tractable: single-gene, large effect sizes, and biochemically measurable endpoints. The evidence base for their clinical implementation is therefore substantially more mature than for pharmacodynamic phenotypes such as AIWG or treatment response. The latter present particular challenges specific to psychiatry: effect sizes are typically modest, phenotypes are polygenic and clinically heterogeneous (Schizophrenia Working Group of the Psychiatric Genomics Consortium et al., 2014; Wray et al., 2018), and endpoints such as treatment response or weight-gain liability are composite outcomes that lack the biochemical precision of metabolic or toxicity measures (Bousman et al., 2021). These features make the translational path longer and the evidential bar higher than for pharmacokinetic pharmacogenes. The repositioning argument applies to both contexts, but at different stages of the translational pipeline. With this distinction in mind, three domains within psychiatric pharmacogenetics illustrate how this sequential pipeline—from genome-wide discovery through mechanistic characterisation to targeted clinical validation—can operate in practice (Figure 1).

The translational pipeline proceeds through four sequential stages, each with a distinct evidential function. Stage 1 (Genome-wide discovery) establishes statistical associations across the genome without a prior mechanistic hypothesis, generating candidate signals for downstream investigation. Stage 2 (Mechanistic characterisation) interrogates prioritised loci using eQTL analyses, epigenomics, and multi-omics integration to identify functional variants and biological pathways. Stage 3 (Targeted pharmacogenetic validation) subjects pre-specified mechanistic hypotheses to confirmatory testing in adequately powered, independently replicated cohorts—this is the stage at which second-generation candidate gene studies contribute. Stage 4 (Clinical implementation) embeds validated gene-drug relationships into prescribing guidelines, EHR-integrated decision support, and clinical workflows. The critical distinction between Stages 1–2 (discovery) and Stages 3–4 (validation and implementation) determines the inferential weight that can be placed on any given finding: genome-wide associations and preliminary mechanistic data cannot be treated as implementation-ready evidence.

## Antidepressant metabolism: CYP2D6 and CYP2C19

CYP2D6 and CYP2C19 were initially identified as pharmacogenetically relevant through biochemical reasoning: as principal phase I oxidative enzymes for most antidepressants, they were biologically obvious candidates. Early candidate gene studies produced inconsistent findings across populations and study designs. The subsequent integration of large-scale genomic data, eQTL analyses, and multi-cohort replication substantially strengthened the evidence base. A meta-analysis pooling individual-level data from 13 clinical studies of European and East Asian populations demonstrated that CYP2D6 and CYP2C19 metaboliser phenotypes significantly predict antidepressant remission, with poor metabolisers at increased risk of adverse drug reactions and ultrarapid metabolisers susceptible to subtherapeutic drug exposure (Milosavljevic et al., 2024; Mrazek et al., 2011). These findings have been incorporated into CPIC guidelines (Hicks et al., 2017; Relling and Klein, 2011). This trajectory closely mirrors the cardiology CYP2C19-clopidogrel story: in both cases, candidate gene reasoning identified the relevant metabolic pathway; underpowered early studies produced inconsistent findings; and guideline implementation followed only after large-cohort replication confirmed clinically meaningful effect sizes. The shared enzyme means that regulatory and implementation frameworks built in cardiology directly strengthen the case for CYP2C19-guided prescribing in psychiatry, a concrete example of how advances in one specialty can accelerate adoption in another.

## Antipsychotic-induced weight gain (AIWG)

Antipsychotic-induced weight gain provides a second informative example in which early candidate gene investigations and subsequent genome-wide analyses converged on overlapping biological targets. Candidate studies identified variants in HTR2C, MC4R, ADRA2A, and DRD2 as potential modulators of metabolic susceptibility, informed by known receptor pharmacology of antipsychotic agents (Zhang et al., 2016). A GWAS subsequently confirmed the MC4R locus as a replicated signal, situating this variant within hypothalamic leptin-melanocortin energy balance networks through post-GWAS multi-omics integration (Locke et al., 2015). Longitudinal validation in a population biobank cohort further substantiated the contribution of polygenic metabolic risk in patients with schizophrenia over a 10-year follow-up (Alver et al., 2024). The AIWG pipeline parallels the oncology experience with DPYD and TPMT: in both settings, a candidate-level hypothesis (receptor pharmacology predicts metabolic susceptibility; enzyme deficiency predicts drug toxicity) was validated by genome-wide and multi-omics methods, and the resulting evidence base is advancing toward clinical utility. It must be emphasised that no clinically validated predictive test for AIWG currently exists. The evidence reviewed here represents a promising but incomplete translational trajectory: prospective, multi-cohort replication across ancestral groups and antipsychotic classes is a prerequisite before any formal guideline incorporation or clinical deployment can be considered (Alver et al., 2024). The key remaining step is not implementation, but robust validation—generating the evidence base that could, in principle, support future clinical adoption.

## PDE4B and substance use disorders

A third, emerging example comes from substance use research, where multi-omics analysis identified PDE4B as both a statistically significant GWAS locus and a mechanistically plausible pharmacological target (Hatoum et al., 2025). The PDE4B inhibitor ibudilast has shown early promise in phase II trials for opioid and alcohol use disorder (Burnette et al., 2020; Metz et al., 2017), but prospective clinical validation remains incomplete. This example is included not as evidence of a completed pipeline, but as an illustration of how the framework operates at an earlier translational stage.

## Organisational implementation of the translational pipeline

The translational gap between genomic discovery and clinical pharmacogenomic implementation is not only a scientific problem but also an organisational one (van der Wouden et al., 2017). Oncology and cardiology demonstrate that successful implementation requires dedicated pharmacogenetics units, EHR-integrated clinical decision support, and formal prescriber education; cardiology achieved point-of-care CYP2C19 genotyping by embedding it directly within catheterisation laboratory workflows (Relling and Evans, 2015; Swen et al., 2023). Three levels of structural integration are needed in psychiatry: at the institutional level, formalised collaborative frameworks between genomic research groups and clinical pharmacology units; at the national level, linkage of psychiatric biobanks and electronic health records for longitudinal pharmacogenetic follow-up, as demonstrated by the Estonian Biobank model (Alver et al., 2024); and at the international level, consortium-based replication extended explicitly to pharmacogenetic phenotypes (Sullivan and Geschwind, 2019). Beyond organisational challenges, a critical limitation of the current evidence base is its predominant derivation from European-ancestry populations: CYP2C19 poor metaboliser alleles are considerably more prevalent in East and South Asian populations. In contrast, CYP2D6 ultrarapid metaboliser phenotypes show markedly higher frequencies in North African populations (Fricke-Galindo et al., 2016; Koopmans, 2021), with direct implications for the generalisability of current guidelines and for equity in precision psychiatry implementation. Future pharmacogenetic research and implementation programmes must prioritise ancestrally diverse cohorts to ensure that clinical benefits are not concentrated in already-advantaged populations.

## Reframed targeted genetic approaches: implications for clinical adoption

An important caveat deserves acknowledgement: pharmacogenetic testing is not uniformly positive. The PRIME Care pragmatic randomised trial found only modest effects on remission rates between pharmacogenomic-guided and usual care groups at 6 months (Oslin et al., 2022), and the clinical utility of commercial pharmacogenetic panels remains contested (Bousman et al., 2021). This underscores a critical distinction: the evidence base supporting specific, well-validated gene-drug pairs such as CYP2D6/CYP2C19 for antidepressant dosing is substantially stronger than that for broader panel-based approaches (Milosavljevic et al., 2024; Mrazek et al., 2011). The repositioning argument advanced here applies to the former, not to undifferentiated multi-gene testing.

Polygenic risk scores represent a parallel and complementary framework for leveraging GWAS data in clinical pharmacogenetic prediction. PRS approaches are actively being developed for antidepressant response, where higher MDD-PRS has been associated with increased odds of non-remission (OR = 1.14 per SD increase) across six European trials (Fanelli et al., 2022). For metabolic risk in schizophrenia, PRS for BMI, schizophrenia, and type 2 diabetes have shown significant associations with AIWG (β = 3.78, 5.38, and 13.4, respectively) (Franz et al., 2024). This framework is not in competition with PRS-based prediction; rather, targeted validation of specific gene-drug pairs addresses the proximate, high-effect-size end of the pharmacogenetic spectrum, while PRS captures the broader polygenic background. A comprehensive precision psychiatry framework will require both.

Within this broader framework, the role of candidate gene studies warrants reconsideration. Their failure as primary discovery tools reflected methodological constraints and a mismatch with polygenic disease architecture (Johnson et al., 2017; Farrell et al., 2015). However, once genome-wide analyses delineate risk landscapes and multi-omics approaches prioritise functional nodes, focused candidate investigations can serve a different purpose: interrogating specific pathways, validating mechanistic hypotheses, and examining gene-treatment interactions within well-characterised clinical cohorts (Trkulja, 2024; Auwerx et al., 2022). This repositioning is not unique to psychiatry: cardiology moved from exploratory candidate pharmacogenetics (CYP2C19 in isolation) to genome-informed targeted validation (POPular Genetics RCT), achieving clinical implementation. Oncology operationalised DPYD through exactly this pathway: a biochemically obvious candidate, confirmed at genome-wide scale, validated prospectively, and embedded in mandatory guidelines. The evidence base for CYP2D6 and CYP2C19 in antidepressant metabolism represents the most mature pharmacogenetic foundation currently available in psychiatry; CPIC guidelines are in place, yet real-world implementation remains variable and the overall clinical utility of pharmacogenetic-guided prescribing is still under active evaluation. For pharmacodynamic phenotypes such as AIWG, prospective multi-cohort replication remains the critical remaining step.

## Conclusion

The future progress of psychiatric genetics will depend on strategic integration: genome-wide analyses to map distributed risk, sequencing to capture rare and structural variation, multi-omics approaches to resolve regulatory mechanisms, and focused candidate investigations to test therapeutic relevance. Within this layered framework, candidate gene studies occupy a narrower but potentially more precise role: no longer engines of discovery, but instruments of translational refinement. Repositioned rather than discarded, candidate gene approaches may contribute to precision psychiatry not by challenging the polygenic paradigm, but by operating within it. The comparative experience of oncology, cardiology, and rheumatology demonstrates that this repositioning is not only theoretically defensible but practically achievable. In each specialty, pharmacogenetic implementation followed the same sequential logic: candidate hypothesis, genome-wide confirmation, prospective clinical validation, and guideline integration. The infrastructure developed in each specialty subsequently provided an organisational and regulatory model for the next. For psychiatric pharmacogenetics, the pharmacokinetic pharmacogene evidence base, specifically CYP2D6 and CYP2C19 in antidepressant metabolism, has reached the stage of guideline incorporation, though uniform clinical implementation remains an ongoing challenge. The remaining challenge is not conceptual but translational: building the clinical workflows, biobank infrastructure, and prescriber education that will allow genomic insights to reach patients across diverse populations.
